# Prenatal diagnosis of 1p34.3 interstitial microdeletion by aCGH in a fetus with jaw bone abnormalities

**DOI:** 10.1186/s13039-016-0288-y

**Published:** 2016-10-06

**Authors:** Themistoklis Dagklis, Elena Papageorgiou, Elisavet Siomou, Vassilis Paspaliaris, Christina Zerva, Panagiotis Chatzis, Loretta Thomaidis, Emmanouil Manolakos, Ioannis Papoulidis

**Affiliations:** 1 Access To Genome - ATG P.C, Clinical Laboratory Genetics, 33A Ethn. Antistaseos str, 55134 Thessaloniki, Greece; 2 Access To Genome - ATG P.C, Clinical Laboratory Genetics, 8 Sisini str, 11528 Athens, Greece; 3 3rd Department of Obstetrics and Gynecology, Aristotle University of Thessaloniki, Hippokration Hospital, Thessaloniki, Greece; 4 Embryomitriki, Prenatal Diagnostic Center, Thessaloniki, Greece; 5 2nd Department of Obstetrics and Gynecology, Aristotle University of Thessaloniki, Hippokration Hospital, Thessaloniki, Greece; 6 Developmental assessment unit, 2nd department of pediatrics, P. & A. Kyriakou children’s hospital, School of medicine, National and Kapodistrian University of Athens, Athens, Greece; 7 Department of Medical Genetics, University of Cagliari, Binaghi Hospital, Cagliari, Italy

**Keywords:** Array-based Comparative Genomic Hybridization array (a-CGH), Chromosome 1, Genotype-phenotype correlation, Microdeletions, Prenatal diagnosis

## Abstract

**Background:**

Interstitial microdeletions in 1p are extremely rare, as very few cases have been reported postnatally and only one prenatally, yet. There is a variability of phenotypic findings such as hypotonia, facial dysmorphisms, mild microcephaly, with being most common developmental delay.

**Case presentation:**

The present case involved a female fetus with an interstitial deletion on 1p, presenting with micrognathia in the 2nd trimester routine ultrasound examination. Array-based comparative genomic hybridization (a-CGH) revealed a 2,7 Mb deletion located on 1p34.3 which could not be detected by standard karyotyping.

**Conclusions:**

This is the first prenatal case of an interstitial deletion in 1p34.3 with facial dysmorphism detected by a-CGH. Due to the use of a-CGH techniques submicroscopic imbalances could be detected, and a refined genotype-phenotype correlation could be achieved.

## Background

Array-based comparative genomic hybridization (array CGH) is a powerful method that allows the detection of submicroscopic alterations in human genome and thus identifies underlying genetic causes that may contribute to various phenotypic abnormalities. On the short arm of chromosome 1, a subtelomeric microdeletion on 1p36 has been well established [1], but interstitial microdeletions in chromosome 1p have rarely been reported. Likewise, some efforts have been made to correlate submicroscopic deletions in 1p34 with a phenotype and such deletions have been associated with dysmorphic features and severe developmental delay [2, 3].

Furthermore, specific genes of this chromosomal region have been associated with distinct phenotypic malformations. More precisely, *GLUT1* deficiency may cause a specific syndrome which correlates with hyperactivity and developmental delay [3, 4], *RIMS3* is considered to be a novel candidate for autism [5], *GRIK3* has also been associated with developmental delay [6], and *AGO1/AGO3* may be responsible for neurocognitive deficits [7]. Furthermore, the chromosomal region 1p34 has been characterized as a tumor suppressor gene locus suggesting a role in cancer development [8].

Until today, there is only 1 case reported in the literature with an interstitial deletion of 1p that was diagnosed prenatally. The deletion spanned the region 1p36.11 to 1p34.3 and was detected by banding cytogenetic method and fluorescence in situ hybridization (FISH) [9]. Here, we report a 2.7 Mb *de novo* interstitial deletion within chromosomal subband 1p34.3, which was diagnosed prenatally in a fetus with micrognathia.

## Case presentation

At 22 weeks of gestation a 34-year-old pregnant female was referred to our lab for prenatal genetic testing after amniocentesis due to the presence of micrognathia detected at the routine 2nd trimester ultrasound examination. The prospective parents were healthy and of Greek origin. This was their first pregnancy and they had no previous medical history. The molecular cytogenetic analysis (see below) revealed a deletion in 1p. Genetic counseling was offered to the couple. At the parents’ request the pregnancy was terminated at 22 weeks of gestation. Subsequently, the female fetus was sent for an autopsy.

### Fetal autopsy

The fetus was of normal growth according to the weeks of gestation (~21/40) weighing 438 g without essential autolytic changes of intrauterine death. The autopsy’s observations are shown in Table 1.

**Table 1:** Autopsy observations of the embryo and the placenta

*Embryo*	*Placenta*
• Cleft palate • Craniofacial malformations (severe posterior micrognathia, microtia) • Narrow trunk with 11 pairs of thoracic and 1 pair of nuchal sides • Abnormal position of fingers • Talipes varus • Knee flexion • Dilatation of fourth ventricle • Malformation of mitral valve	• Underweight placenta with increased fetus-placenta ratio • Mitral decidual arteriopathy • Low-grade acute chorioamniotis maternally derived, without any inflammatory reaction observed in the fetus

The mitral decidual arteriopathy observed in the placenta indicated pathological implantation, which possibly caused uteroplacental insufficiency and relevant gestational complications uteroplacental ischemia. In the present case, however it is possible that the decidual arteriopathy and the underweight placenta just reflect the pathological implantation and the abnormal placentation of a genetically pathological fetus.

### Molecular cytogenetic analysis

Molecular karyotype analysis by array CGH using “Illumina Cytochip Focus Constitutional array with BAC technology” was performed on DNA isolated from uncultured amniocytes according to the manufacturer’s protocol. The Illumina Cytochip Focus Constitutional array is a commercially available whole-genome BAC array with a median resolution of 0.5−1 Mb. (Cytochip Focus Constitutional, Illumina).

The parent’s blood derived DNA samples were prepared from peripheral blood leukocytes (Promega, Madison, WI, USA). Array data was analyzed using Bluefuse software analysis (BlueGnome Ltd., UK) using GRCh37/hg19 UCSC assembly and compared to known duplication listed in public available databases [Database of Genomic Variants (DGV, http://projects.tcag.ca/variation/webcite), ENSEMBL (http://www.ensembl.orgwebcite), and DECIPHER (http://decipher.sanger.ac.ukwebcite) Accessed at 25/01/2015].

**Fig. 1:**
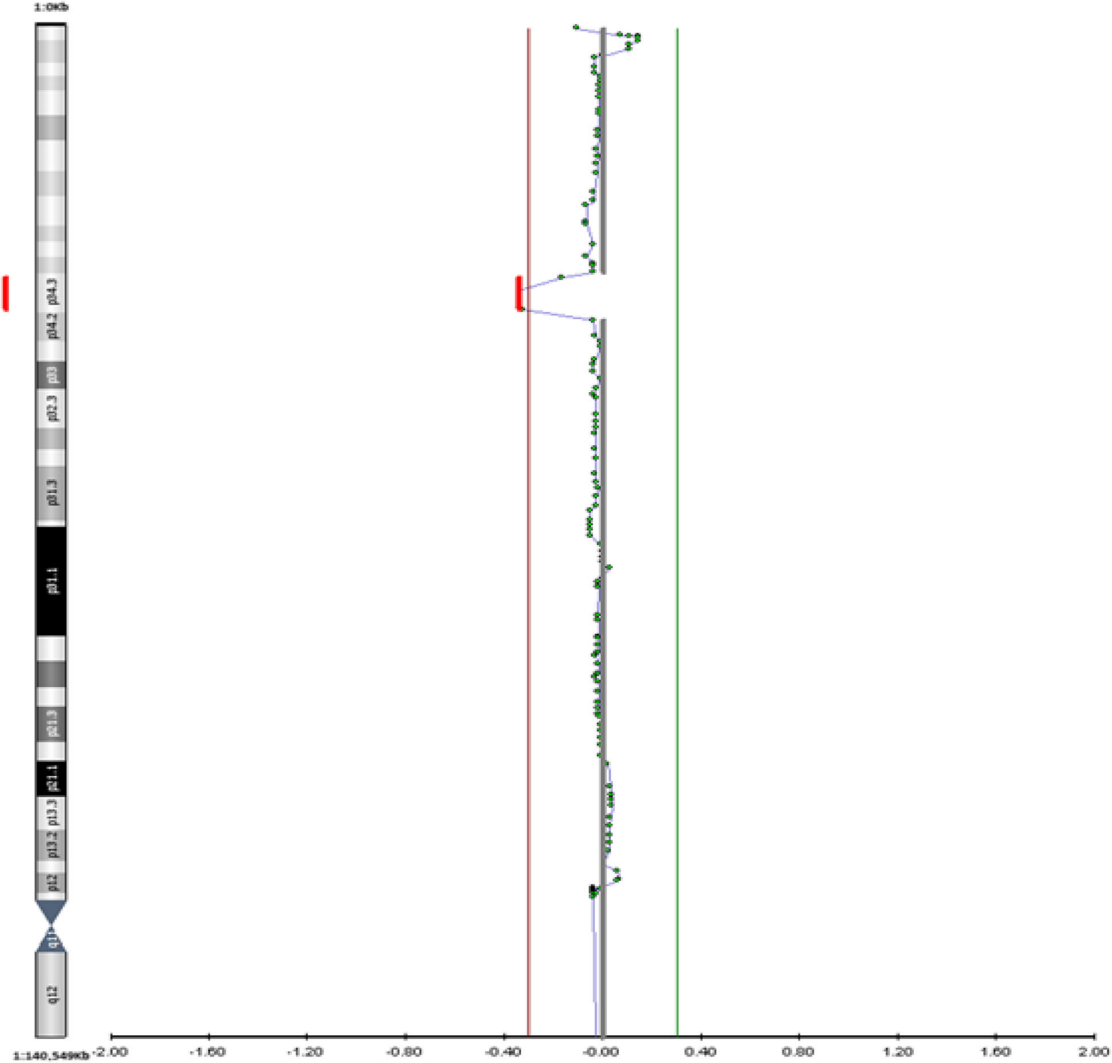
Array-CGH analysis illustrating in depth the *de novo* interstitial microdeletion of approximately 2,7 Mb in size on the short arm of chromosome 1 at chromosomal band 1p34.3 (location: 36,901,642 − 39,606,756 using build GRCh37 (hg19))

A female profile was revealed with a 2,7 Mb deletion at chromosome 1p34.3 extending from position 36,901,642 to 39,606,756 (GRCh37/hg19 Feb.2009) (Fig. 1). No other copy number variant was detected at the referred sample. Using the UCSC Genome Browser and the OMIM database we observed that the deleted region contains 27 OMIM genes, listed in Table 2. Parental blood testing with a-CGH method revealed that the deletion occured *de novo.*

**Table 2:** OMIM genes included in the deleted region

Gene symbol	OMIM number
*OSCP1*	608854
*MRPS15*	611979
*CSF3R*	138971
*GRIK3*	138243
*ZC3H12A*	610562
*MEAF6*	611001
*SNIP1*	608241
*DNALI1*	602135
*GNL2*	609365
*RSPO1*	609595
*C1orf109*	614799
*CDCA8*	609977
*EPHA10*	611123
*YRDC*	612276
*MTF1*	600172
*INPP5B*	147264
*SF3A3*	605596
*FHL3*	602790
*UTP11L*	609440
*POU3F1*	602479
*RRAGC*	608267
*MYCBP*	606535
*GJA9*	611923
*RHBDL2*	608962
*AKIRIN1*	615164
*NDUFS5*	603847
*MACF1*	608271

## Discussion

We report a prenatal case, which involves an interstitial microdeletion within chromosome 1p34.3. Most of the cases that have been reported with similar deletions are postnatal and only 1 is a prenatal report [9]. There is no specific phenotype or known syndrome that has been associated to this kind of deletion. To our knowledge there are only about 8 comparable patients, and in most cases the detected rearrangement was de novo as in our case. These patients showed a variability of phenotypic findings with developmental delay to be apparently present in almost all of them (Table 3).

**Table 3:** Overview of patients with 1p34.3 deletion. NM: Not Mentioned

	Tokita et al. [7] proband 1	Tokita et al. [7] proband 2	Tokita et al. [7] proband 3	Tokita et al. [7] proband 4	Tokita et al. [7] proband 5	Martinez et al. [2] proband 1	Martinez et al. [2] proband 2	Takenouchi et al. [6]
Gender	F	F	F	M	M	M	F	F
Delivery age (weeks)	38	37	42	41	38	NM	NM	37
Pregnancy and delivery	Uncomplicated	Uncomplicated	Uncomplicated	IUGR, neonatal sepsis	IUGR, perinatal asphyxia	NM	NM	Uncomplicated
Feeding difficulties	No	Yes	Yes	Yes	Yes	No	No	Yes
Age	3y 9 m	10y 6 m	18y	17 m	13y	13y	8y	8y
Height (percentile)	24th	25th	50th	<1st	90th-97th	50th	50th	NM
Weight (percentile)	15th	2nd	5th	1st	>97th	50th	50th	NM
OFC (percentile)	3rd-10th	25th	<<3rd	<1st	2nd-10th	50th	50th	NM
Developmental delay	Yes	Yes	Yes	Yes	Yes	No	No	Yes
Facial deformities	Yes	Yes	Yes	Yes	Yes	Yes	No	No
Hypotonia	Yes	Yes	Yes	Yes	Yes	No	No	No

Takenounci et al. [6] reported about a young girl, showing severe developmental delay, mild retrognathia and slightly downslanting papebral fissures. The deleted chromosomal regions in this proband and in the present case encompass the *GRIK3* gene which was suggested to be responsible for neurodevelopmental manifestations by Takenounci et al. [6].

Another gene that might contribute to craniofacial malformation and it is haploinsufficient in this case is *SNIP1*. Puffenberger et al. [10] identified a homozygous 1097A-G transition in the *SNIP1* gene in 3 Amish patients with severe craniofacial dysmorphism. Western blot analysis showed decreased levels of the mutant homologous murine protein, suggesting that it is unstable. Puffenberger et al. [10] postulated that decreased abundance of SNIP1 likely affects c-Myc activity, TGF-beta, and NF-kappa-B signaling, as SNIP1 protein interacts with these pathways [11–13], and this effect may cause abnormal brain and skull development.

**Fig. 2:**
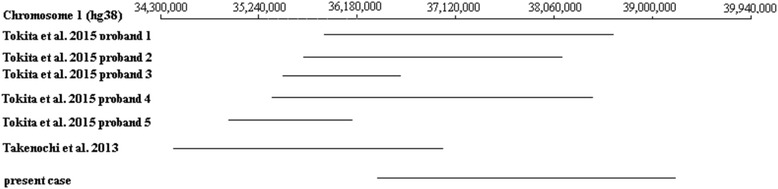
Schematic presentation of 1p34.3 deletions

Recent data from Tokita et al. [7] describe five children with microdeletions on 1p34.3 that showed, apart from hypotonia and developmental delay, craniofacial dysmorphisms such as retrognathia and small ears, as well feet and fingers malformations. The deletion that we report here overlaps with 4 deleted chromosomal loci, except a region between 38,622,840–39,141,084 (Fig. 2). In this region there is the 5’ end of *MACF1* gene (chr1:39,084,166–39,487,137). MACF1 protein was found to be greatly up-regulated upon differentiation of myoblasts into myotubes [14], while Kodama et al. [15] showed that mouse *MACF1* (or *Acf7*) is an essential integrator of microtubule-actin dynamics. In the absence of MACF1 the consequences were long, less stable microtubules with skewed cytoplasmic trajectories and altered dynamic instability [15]. Taking these into account it might be explained the fact that the present case exhibited dilatation of fourth ventricle and malformation of mitral valve, while none of the cases of Table 3 presented any heart defect.

The fetal autopsy also showed pathological implantation of the fetus, and a possible explanation is that the placenta exhibited abnormal extracellular matrix. Ephrins are membrane-bound proteins that interact with Eph receptors, and have a crucial role in many developmental processes like guidance of neuron axon growth cones, cell migration and formation of tissue boundaries [16]. In the present case, 1 Eph receptor, *EPHA10*, is haploinsufficient, and is possibly 1 of the causes of improper implantation of the fetus, due to abnormal interaction of placental and uterus tissue boundaries.

## Conclusion

Array-based comparative genomic hybridization has been placed in the routine prenatal genetic testing and novel microscopic imbalances through the genome come to light. The comparison with similar findings in other patients is still not possible in most of the cases, but collecting data from such cases is crucial for genetic counselors to interpret prenatal cases, and achieve more accurate reports about clinical manifestations.

## Abbreviations

aCGH: Array comparative genomic hybridization
OFC: Orbito-frontal cortex

## References

[1] Shimada S, Shimojima K, Okamoto N, Sangu N, Hirasawa K, Matsuo M, Ikeuchi M, Shimakawa S, Shimizu K, Mizuno S, Kubota M, Adachi M, Saito Y, Tomiwa K, Haginoya K, Numabe H, Kako Y, Hayashi A, Sakamoto H, Hiraki Y, Minami K, Takemoto K, Watanabe K, Miura K, Chiyonobu T, Kumada T, Imai K, Maegaki Y, Nagata S, Kosaki K, Izumi T, Nagai T, Yamamoto T (2015). Microarray analysis of 50 patients reveals the critical chromosomal regions responsible for 1p36 deletion syndrome-related complications. Brain Dev.

[2] Martinez JE, Tuck-Muller CM, Gasparrini W, Li S, Wertelecki W (1999). 1p microdeletion in sibs with minimal phenotypic manifestations. Am J Med Genet.

[3] Vermeer S, Koolen DA, Visser G, Brackel HJ, van der Burgt I, de Leeuw N, Willemsen MA, Sistermans EA, Pfundt R, de Vries BB (2007). A novel microdeletion in 1(p34.2p34.3), involving the SLC2A1 (GLUT1) gene, and severe delayed development. Dev Med Child Neurol.

[4] Aktas D, Utine EG, Mrasek K, Weise A, von Eggeling F, Yalaz K, Posorski N, Akarsu N, Alikasifoglu M, Liehr T, Tuncbilek E (2010). Derivative chromosome 1 and GLUT1 deficiency syndrome in a sibling pair. Mol Cytogenet.

[5] Kumar RA, Sudi J, Babatz TD, Brune CW, Oswald D, Yen M, Nowak NJ, Cook EH, Christian SL, Dobyns WB (2010). A de novo 1p34.2 microdeletion identifies the synaptic vesicle gene RIMS3 as a novel candidate for autism. J Med Genet.

[6] Takenouchi T, Hashida N, Torii C, Kosaki R, Takahashi T, Kosaki K (2014). 1p34.3 deletion involving GRIK3: Further clinical implication of GRIK family glutamate receptors in the pathogenesis of developmental delay. Am J Med Genet A.

[7] Tokita MJ, Chow PM, Mirzaa G, Dikow N, Maas B, Isidor B, Le Caignec C, Penney LS, Mazzotta G, Bernardini L, Filippi T, Battaglia A, Donti E, Earl D, Prontera P (2015). Five children with deletions of 1p34.3 encompassing AGO1 and AGO3. Eur J Hum Genet.

[8] Sulman EP, White PS, Brodeur GM (2004). Genomic annotation of the meningioma tumor suppressor locus on chromosome 1p34. Oncogene.

[9] Yang H, Lee CL, Young DC, Shortliffe M, Yu W, Wright JR (2004). A rare case of interstitial del(1)(p34.3p36.11) diagnosed prenatally. Fetal Pediatr Pathol.

[10] Puffenberger EG, Jinks RN, Sougnez C, Cibulskis K, Willert RA, Achilly NP, Cassidy RP, Fiorentini CJ, Heiken KF, Lawrence JJ, Mahoney MH, Miller CJ (2012). Genetic mapping and exome sequencing identify variants associated with five novel diseases. PLoS ONE.

[11] Fujii M, Lyakh LA, Bracken CP, Fukuoka J, Hayakawa M, Tsukiyama T, Soll SJ, Harris M, Rocha S, Roche KC, Tominaga S, Jen J, Perkins ND, Lechleider RJ, Roberts AB (2006). SNIP1 is a candidate modifier of the transcriptional activity of c-Myc on E box-dependent target genes. Molec Cell.

[12] Kim RH, Flanders KC, Reffey SB, Anderson LA, Duckett CS, Perkins ND, Roberts AB (2001). SNIP1 inhibits NF-kappa-B signaling by competing for its binding to the C/H1 domain of CBP/p300 transcriptional co-activators. J Biol Chem.

[13] Kim RH, Wang D, Tsang M, Martin J, Huff C, de Caestecker MP, Parks WT, Meng X, Lechleider RJ, Wang T, Roberts AB (2000). A novel Smad nuclear interacting protein, SNIP1, suppresses p300-dependent TGF-beta signal transduction. Genes Dev.

[14] Sun Y, Zhang J, Kraeft SK, Auclair D, Chang MS, Liu Y, Sutherland R, Salgia R, Griffin JD, Ferland LH, Chen LB (1999). Molecular cloning and characterization of human trabeculin-alpha, a giant protein defining a new family of actin-binding proteins. J Biol Chem.

[15] Kodama A, Karakesisoglou I, Wong E, Vaezi A, Fuchs E (2003). ACF7: an essential integrator of microtubule dynamics. Cell.

[16] Davy A, Soriano P (2005). Ephrin signaling in vivo: look both ways. Dev Dyn.

